# Enhanced virulence and neuroinvasion of contemporary *Oropouche* virus strains in the AG129 mouse model

**DOI:** 10.3389/fmicb.2026.1771021

**Published:** 2026-05-19

**Authors:** Ellen Caroline Nobre Santos, Nathália Costa Silva, Luiza Godoy Rodrigues Pinto, Luiz Gustavo Pontes Santos, Juliana da Silva Brandi Oliveira, Melissa Teixeira Goulart, Marcele Neves Rocha, Isaque João da Silva de Faria, Renan Pedra de Souza, Silvana F. de Mendonça, Livia Baldon, Yaovi M. H. Todjiro, Amanda C. de Freitas, Rafaela L. Moreira, Maria E. C. Rodrigues, Siad Amadou, José M. G. Fernandes, Natália R. Guimarães, Talita E. R. Adelino, Sara C. F. dos Santos, Felipe C. M. Iani, Keldenn M. F. Moreno, João P. P. de Almeida, Luiz Alcantara, Marta Giovanetti, Fabíola Mara Ribeiro, Álvaro G. A. Ferreira

**Affiliations:** 1 Mosquitos Vetores, Endossimbiontes e Interação Patógeno-Vetor, Instituto René Rachou-Fiocruz, Belo Horizonte, Brazil; 2 Departamento de Bioquímica e Imunologia, Instituto de Ciências Biológicas, Universidade Federal de Minas Gerais, Belo Horizonte, Brazil; 3 Departamento de Genética, Ecologia e Evolução, Instituto de Ciências Biológicas, Universidade Federal de Minas Gerais, Belo Horizonte, Brazil; 4 Departamento de Bioinformática, Instituto de Ciências Biológicas, Universidade Federal de Minas Gerais, Belo Horizonte, Brazil; 5 Fundação Ezequiel Dias, Belo Horizonte, Brazil; 6 Oswaldo Cruz Institute, Oswaldo Cruz Foundation, Rio de Janeiro, Brazil; 7 Department of Science and Bio-Technology, Universita Campus Bio-Medico di Roma, Rome, Italy

**Keywords:** Oropouche virus, AG129 strain mice, neuroinvasion, central nervous system, viremia, tissue tropism, arthropod-borne viroses

## Abstract

Oropouche virus (OROV) is an orthobunyavirus undergoing dramatic re-emergence and rapid geographic expansion across Central and South America, coinciding with the emergence of reassortant lineages carrying novel segment combinations and adaptive mutations. Despite extensive genomic and epidemiological characterization, the biological mechanisms driving OROV resurgence during 2023–2024 remain unclear. Here, we show that contemporary reassortant OROV isolates exhibit markedly enhanced virulence in the AG129 mouse model compared with the historical prototype strain OROV_BeAn19991_. Two reassortant 2024 isolates—OROV_SC2024_ from a non-Amazonian region and OROV_AC2024_ from the Amazon Basin—produced significantly higher viremia, accelerated disease progression, and increased mortality. These isolates disseminated widely, generating substantially elevated viral loads across multiple organs, including the spleen, kidney, liver, lung, uterus, and testes, and—most notably—demonstrated a heightened capacity for the brain. Infection with contemporary reassortant lineages also induced pro-inflammatory responses, characterized by consistent upregulation of IL-1β across tissues. Together, these findings provide experimental evidence that ongoing viral evolution is likely driving a measurable increase in OROV pathogenicity, providing a biological framework to better understand the increased severity of clinical cases reported in recent outbreaks. This work underscores the urgent need for enhanced surveillance, real-time genomic monitoring, and development of targeted countermeasures against this rapidly spreading arbovirus.

## 1 Introduction

Arthropod-borne viruses (arboviruses) account for a large proportion of emerging and re-emerging infectious diseases, imposing substantial global health and socioeconomic burdens (Weaver and Barrett, 2004; Wilder-Smith et al., 2017). Over recent decades, the reintroduction, ecological expansion, and sustained transmission of viruses such as dengue (DENV), West Nile (WNV), Chikungunya (CHIKV), and Zika (ZIKV) have demonstrated the capacity of arboviruses to shift rapidly from localized circulation to widespread epidemics (Mayer et al., 2017; Weaver et al., 2018). These events underscore how ecological, demographic, and socioeconomic changes—combined with viral evolution—can enhance transmissibility, virulence, and disease severity (Bartlett et al., 2025; de Souza et al., 2025).

Oropouche virus (OROV; *Orthobunyavirus oropoucheense*) is a striking recent example of an arbovirus undergoing rapid geographic and epidemiological expansion. A member of the family *Peribunyaviridae*, the OROV genome consists of three negative-sense RNA segments: the L segment, encoding the RNA-dependent RNA polymerase; the M segment, encoding the Gn and Gc glycoproteins and the non-structural protein M (NSm); and the S segment, encoding the nucleocapsid (N) and the non-structural protein S (NSs) (Elliott, 2014; Hughes et al., 2020). Since its first detection in Trinidad and Tobago in 1955, OROV has caused multiple explosive outbreaks, historically concentrated in the Amazon Basin, where it circulates in a sylvatic cycle involving sloths, non-human primates, and birds, with human transmission predominantly mediated by the biting midge *Culicoides paraensis* (Anderson et al., 1961; Pinheiro et al., 1962, 1981, 1982; Tesh, 1994; Romero-Alvarez and Escobar, 2018). Although detected in mosquitoes such as *Culex quinquefasciatus*, their epidemiological relevance appears limited (de Mendonça et al., 2021, 2025).

Clinically, OROV typically causes an acute febrile illness that is often self-limiting; however, neurological complications, including aseptic meningoencephalitis, have been documented, and recent outbreaks have revealed a broader clinical spectrum than previously recognized (Vasconcelos et al., 1989; Tilston-Lunel, 2024). Importantly, despite being historically restricted to Amazonian regions of north of Brazil, OROV has undergone a rapid and unprecedented geographic expansion in recent years. Brazil has reported outbreaks across multiple non-Amazonian states, demonstrating sustained autochthonous transmission well beyond its traditional ecological niche and now encompassing all five Brazilian macro-regions (North, Northeast, Southeast, South, and Midwest) (Figueiredo, 2007; Lima-Camara, 2016; Gutierrez et al., 2020; Scachetti et al., 2024; Gräf et al., 2025). Concurrently, increasing numbers of infections have been reported across Latin America, with confirmed circulation in Peru, Ecuador, Colombia, Venezuela, Bolivia, Panama, Guyana, and, more recently, Cuba, alongside travel-associated cases identified in Europe (Forshey et al., 2010; Navarro et al., 2016; Wise et al., 2020; Durango-Chavez et al., 2022; Gil-Mora et al., 2022; Castilletti et al., 2024; Deiana et al., 2024; Wesselmann et al., 2024; de Melo Iani et al., 2025). Several ecological and environmental forces are likely contributing to OROV’s recent expansion. Large-scale landscape disturbances—such as deforestation, habitat fragmentation, agricultural intensification, and accelerated peri-urban growth—may have reshaped the distribution and abundance of *Culicoides paraensis*, increasing opportunities for human–vector contact. Climate anomalies linked to global warming and altered rainfall patterns could further prolong vector activity and enhance survival.

Beyond these ecological and anthropogenic drivers, genomic analyses from recent outbreaks reveal the emergence and widespread circulation of reassortant OROV lineages carrying novel segment combinations and adaptive mutations (Gutierrez et al., 2020; Moreira et al., 2024; Naveca et al., 2024; Scachetti et al., 2024; de Melo Iani et al., 2025). Such genomic changes are hypothesized to modulate key phenotypic traits, including replication dynamics, tissue tropism, and host immune antagonism, potentially altering viral fitness. Notably, the dominance of these molecular signatures has coincided with reports of severe clinical presentations, including neurological complications and the first documented fatalities in Brazil (Bandeira et al., 2024; Dashraath et al., 2024; Filho et al., 2024a, 2024b; Martins-Filho et al., 2024; Sah et al., 2024; Schwartz et al., 2024; de Melo Iani et al., 2025; Brazilian Institute of Geography and Statistics (IBGE), 2020). While increased clinical reporting may be influenced by enhanced surveillance, these observations warrant an experimental evaluation of whether currently circulating OROV lineages exhibit altered intrinsic pathogenicity. Testing this hypothesis is essential to determine how ongoing viral evolution contributes to disease severity and to assess the risk that continued adaptation may further amplify the public health impact of this neglected arbovirus.

Here, to test this hypothesis, we investigated OROV infection dynamics and pathogenesis in a vertebrate host using the AG129 mouse (IFN *α*/*β*/*γ* R^−/−^) model, directly comparing the historical reference strain OROV_BeAn19991_ with two highly circulating 2024 strains bearing novel reassortments and adaptive mutations—OROV_SC2024_, isolated from a non-Amazon region, and OROV_AC2024_, isolated from the Amazon Basin. Our results demonstrate that these contemporary reassortment lineages produced markedly higher viremia levels and induced significantly greater mortality in mice, indicating enhanced pathogenicity relative to the historical strain. Moreover, mice infected with contemporary isolates exhibited increased viral loads in nearly all tissues examined—including spleen, kidney, liver, lung, uterus, testes, and brain—alongside a consistent trend toward elevated IL-1β expression across most organs.

## 2 Materials and methods

### 2.1 Production of viral stock and titration

#### 2.1.1 OROV strains

**Figure 1:**
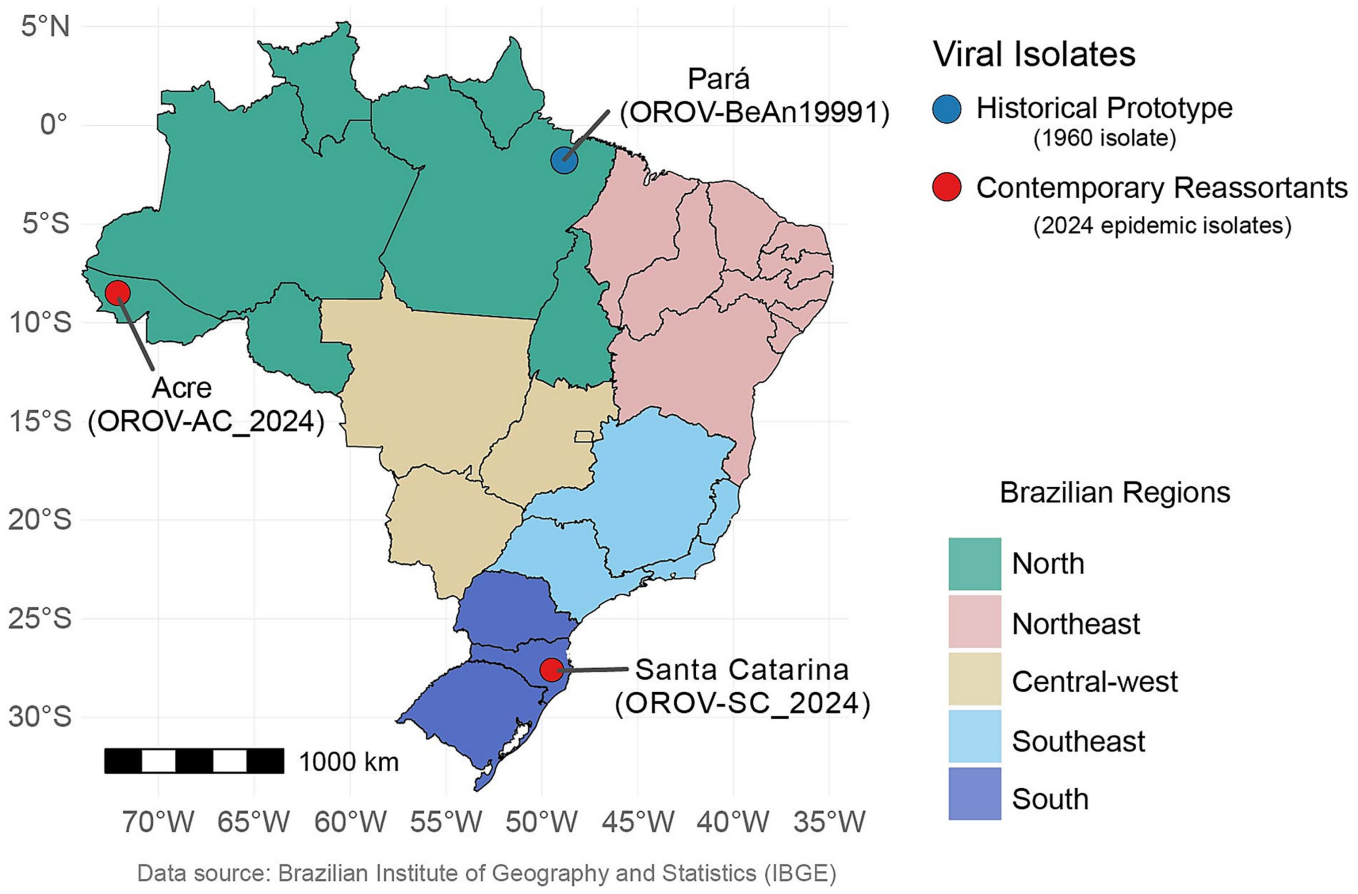
Geographic origin of OROV isolates. Geographic map of Brazil demonstrating the spatial distribution of the contemporary and ancestral OROV strains used in this study. The map highlights the five Brazilian macro-regions by color to contextualize the phylogenetic lineages and geographical expansion. Ancestral strain (OROV_BeAn19991_): isolated near the Belém-Brasília highway in Pará (North region) from the blood of captured sloth (*Bradypus tridactylus*) (Pinheiro et al., 2002). Contemporary strains: OROV_AC2024_ was isolated from a human patient in Cruzeiro do Sul, Acre (North region), and OROV_SC2024_ was isolated from human patient in Botuverá, Santa Catarina (South region) (de Melo Iani et al., 2025). Maps were generated in R software using the geobr package (Pereira et al., 2019) with data adapted from the Brazilian Institute of Geography and Statistics (Brazilian Institute of Geography and Statistics (IBGE) 2020).

This investigation utilized three distinct OROV strains. The reference strain, OROV prototype (BeAn19991), was initially isolated in Brazil’s Amazon region from the blood of a three-toed sloth (*Bradypus tridactylus*). Its segmented genome sequences are accessible via GenBank under accession numbers KP052850 (L segment), KP052851.1 (M segment), and KP052852 (S segment) (Pinheiro et al., 2002; Tilston-Lunel et al., 2015). In addition to the prototype, two recent human clinical isolates were included: OROV_SC2024_, obtained from the serum of a febrile patient in Botuverá, Santa Catarina (SC), Brazil, in early 2024 (GenBank: PQ247721, PQ168305, and PQ168436); and OROV_AC2024_, isolated from a febrile individual’s serum in Cruzeiro do Sul, Acre (AC), Brazil, in 2024 (GenBank: PQ247716, PQ168300, and PQ168431) (Figure 1) (Pereira et al., 2019).

#### 2.1.2 Cell maintenance and virus production

The C6/36 cell line, derived from *Aedes albopictus* mosquitoes, was utilized as the cellular substrate for OROV propagation. These cells were grown in Leibovitz’s L15 Medium (L15, Sigma-Aldrich, St. Louis, MO, USA) supplemented with 10% Fetal Bovine Serum (FBS, Sigma-Aldrich, St. Louis, MO, USA) and a 1x Antibiotic-Antimycotic solution (Sigma-Aldrich, St. Louis, MO, USA). Cultures were maintained at 28 °C within a Biochemical Oxygen Demand (BOD) incubator. For large-scale virus amplification, cells were seeded into 25 or 75 cm^2^ flasks (Sarstedt, Nümbrecht, Germany) to achieve 70% confluence prior to infection. Infection was carried out using the respective OROV strains at a Multiplicity of Infection (MOI) of 0.01. Following the 1-h viral adsorption phase at 28 °C, L15 maintenance medium was added directly to the flask to achieve a final volume of 5 mL. The infected cultures were then incubated for a total duration of 6 to 7 days at 28 °C. The cell culture supernatant was subsequently collected. These supernatants were then clarified by centrifugation at 3,000 × *g* for 15 min to effectively pellet any cellular debris. Purified viral aliquots were stored at −80 °C.

#### 2.1.3 Plaque assay for virus titration

The infectivity of the viral stocks was determined on Vero cells (African green monkey kidney cells) using the plaque-forming unit (PFU) assay. Vero cells were maintained in DMEM-High medium supplemented with 10% FBS and 1% Penicillin/Streptomycin at 37 °C with 5% CO_2_. For titration, confluent Vero monolayers in six or twelve-well plates were inoculated with serially diluted virus preparations. The adsorption process was performed for 1-h at 37 °C in 5% CO_2_, with intermittent rocking. The plates were then overlaid with a semi-solid medium containing 1.5% Carboxymethylcellulose (CMC) (Synth, São Paulo, Brazil) diluted in DMEM-Low (supplemented with 5% FBS and 1% Penicillin/Streptomycin). Plaque development was allowed over 5 days of incubation. After this period, cell monolayers were fixed with 3.7% formaldehyde. The CMC overlay was removed by washing, and visible plaques were stained using a Crystal Violet solution 0.25% crystal violet solution (in a 70% water/30% ethanol mixture) for approximately 1-h. Following final washes and drying, the resulting lysis plaques were counted, and the viral titer was expressed as PFU/mL.

### 2.2 Mice lineage

For the *in vivo* component of this investigation, we utilized the AG129 mouse lineage (IFN- *α*, *β*, *γ* R^−/−^). This double knockout immunocompromised model is characterized by the absence of receptors for both Type I Interferon (IFN-α, β) and Type II Interferon (IFN-γ), rendering the animals highly susceptible to viral challenge (Baldon et al., 2022). The AG129 mice were sourced from and maintained within the breeding colony at the Animal Facility of the Instituto René Rachou, Fiocruz Minas. All animal procedures were rigorously reviewed and formally approved by the local Institutional Animal Care and Use Committee (Comissão de Ética no Uso de Animais da Fiocruz - CEUA). Experiments were executed in strict accordance with institutional guidelines, under the established protocol and license number LW-28-25.

### 2.3 OROV inoculation and viremia kinetics in AG129 mice

Inoculation of the AG129 mice was performed via the intraperitoneal (IP) route. For OROV studies, four-week-old animals were utilized, and each animal received an approximate dose of 10^4^ plaque-forming units (PFU). Following inoculation, mice were monitored daily to assess morbidity and mortality. To determine viremia kinetics, 0.2 mL blood samples were collected from individual mice at specific time points. Blood was consistently drawn from the tail vein after the animals were briefly anesthetized. All AG129 mice used in the inoculation experiments were bred and housed in a Specific-Pathogen-Free (SPF) facility at the Instituto René Rachou. The animals were maintained in an environment with controlled temperature and humidity, operating on a 12-h light/dark cycle. Food and water were provided *ad libitum* throughout the duration of the experiments.

### 2.4 Tissue collection and processing

At the pre-defined experimental endpoint, mice were deeply anesthetized via intraperitoneal injection of ketamine (80 mg/kg) and xylazine (8 mg/kg) and transcardially perfused with phosphate-buffered saline (PBS, 1×) to clear circulating blood. Immediately after perfusion, organs were harvested. The collected tissues included key central nervous system (CNS) regions (striatum, hippocampus, cortex, and cerebellum), alongside several peripheral organs (lung, liver, spleen, kidney, and either testis or uterus). All collected organs were promptly stored for subsequent RNA extraction. This procedure was essential for quantifying the viral RNA and definitively verifying the presence and dissemination of the infection in each specific tissue.

### 2.5 Viral RNA extraction and quantitative real-time PCR

#### 2.5.1 RNA extraction from tissues

To assess viral load within the collected tissues (striatum, hippocampus, cortex, cerebellum, lung, liver, spleen, kidney, testis/uterus), total RNA was isolated. The harvested organs were mechanically homogenized in TRIzol reagent (Invitrogen) using glass beads. Total RNA was subsequently extracted from the homogenized samples following the manufacturer’s instructions.

#### 2.5.2 cDNA synthesis (reverse transcription)

Total RNA concentration was determined using a NanoDrop Multiskan GO spectrophotometer. A mass of 1,000 ng of total RNA was used as a template for the synthesis of the first-strand cDNA. The reaction was performed using M-MLV Reverse Transcriptase (Promega®, Madison, WI, USA) and Random Primers in a final volume of 20 μL, containing 625 μM dNTPs and 0.01 M DTT.

Initially, the RNA and random primers were subjected to denaturation at 70 °C for 10 min, followed by immediate chilling (cooling to 4 °C). The M-MLV enzyme (2 units) and reaction buffer components were then added, and the synthesis was incubated at 42 °C for 60 min. Enzyme inactivation was performed at 72 °C for 5 min. The resulting cDNA was stored at −20 °C.

#### 2.5.3 Quantitative real-time PCR

The viral RNA load in the cDNA samples was quantified using quantitative Real-Time Polymerase Chain Reaction (RT-qPCR). The reactions were performed utilizing the Power SYBR Green Master Mix (Applied Biosystems—Life Technologies, Foster City, CA, USA), strictly adhering to the manufacturer’s protocol.

The OROV viral load was determined relative to the expression of two endogenous housekeeping control genes: RPL32 (ribosomal protein L32) and HPRT (Hypoxanthine Phosphoribosyl transferase) gene, ensuring robust data normalization across tissue samples.

The sequences for the primers used in the amplification reaction were as follows: the OROV-specific forward primer 5′-CAACGATGTACCACAACGGACTAC-3′ and reverse 5′-ACAACACCA GCATTGAGCACTT-3′. The IL1-*β* primers were forward: 5′-GGGCCTCAAAGGAAAGAATC-3′ and reverse: 5′-TACCAGTTGGGGAACTCTGC-3′. The IL-6 primers were forward: 5′-ACG GCC TTC CCT ACT TCA CA-3′ and reverse: 5′-CAT TTC CAC GAT TTC CCA GA-3′. The TNF-*α* primers were forward: 5′-GCTGAGCTCAAACCCTGGTA-3′ and reverse: 5′-CGGACTCCGCAAAGTCTAAG-3′. The HPRT housekeeping control primers were forward: 5′-TCAGTCAAC GGGGGACATAAA-3′ and reverse: 5′-GGGGCTGTACTGCTTAACCAG-3′. Finally, the RpL32 housekeeping primers were forward: 5′-GCTGCCATCTGTTTTACGG-3′ and reverse: 5′-TGACTGGTGCCTGATGAACT-3′.

All reactions were conducted in a QuantStudio™ 7 Flex Real-Time PCR System (Applied Biosystems) under optimized cycling conditions. The final viral RNA load was calculated using the 2^−ΔΔ*Ct*^ method and expressed as the relative quantification of the OROV genome copies normalized to the RpL32 and HPRT reference genes.

### 2.6 Phylogenetics analysis

Sequences of the S, M, and L genomic segments from the OROV experimental isolates used in this study were combined with corresponding segments from recently published full-length OROV genomes available in NCBI/GenBank, including genomes derived from fatal human cases. Independent datasets were generated for each genomic segment, comprising 379 S segment sequences, 234 M segment sequences, and 306 L segment sequences, all including the reference strain BeAn19991. Sequence alignments for each segment dataset were generated using MAFFT (Katoh and Standley, 2013) and manually curated to remove alignment artefacts using AliView (Larsson, 2014). The full-genome dataset, generated by concatenation of individual segments, was screened for potential recombination and reassortment using RDP5, applying default parameters, except that segments were treated as linear sequences and window sizes of 16, 50, 40, and 101 were used for the RDP, Chimaera, MaxChi, and Bootscan methods, respectively (Martin et al., 2020). Genomic regions identified by RDP5 as recombinant, as well as segments inferred to have been acquired through reassortment, were removed from the alignment by replacing the corresponding positions with gap characters (“–”), yielding a recombination—and reassortment-free full-genome alignment. Both the curated full-genome alignment and individual segment alignments were used to infer maximum-likelihood (ML) phylogenetic trees using IQ-TREE v2 (Minh et al., 2020) under the HKY nucleotide substitution model, as selected by ModelFinder. Branch support was assessed using the approximate likelihood-ratio test based on bootstrap and the Shimodaira–Hasegawa–like procedure with 1,000 replicates.

### 2.7 Statistical analysis

All statistical tests were performed using the R software environment (version 4.3.3). Custom scripts were developed for data processing and graphical generation utilized the ggplot2 package (Wilkinson, 2005; Valero-Mora, 2010). For the analysis of survival data (morbidity and mortality assessment), Kaplan–Meier survival curves were generated, and statistical differences between the experimental groups were determined using the Log-Rank test. The Cox Proportional Hazards Model utilized to quantify the differential virulence (Hazard Ratio, HR) between the strains.

For the analysis of viral load (viremia and tissue measurements), we fitted a Generalized Linear Model (GLM) assuming a Gamma distribution. Pairwise comparisons were performed using the *emmeans* package with Tukey’s adjustment for multiple testing. Viral-load analyses were restricted to detectable measurements only, as quantification is meaningful exclusively for samples with measurable RNA levels. Model assumptions were evaluated using the *DHARMa* package. Infection prevalence was assessed by comparing the proportions of detected versus non-detected viral RNA across strains using Fisher’s exact test. The significance level was set at 0.05.

## 3 Results

### 3.1 Phylogenetic analysis of the OROV lineages

**Figure 2:**
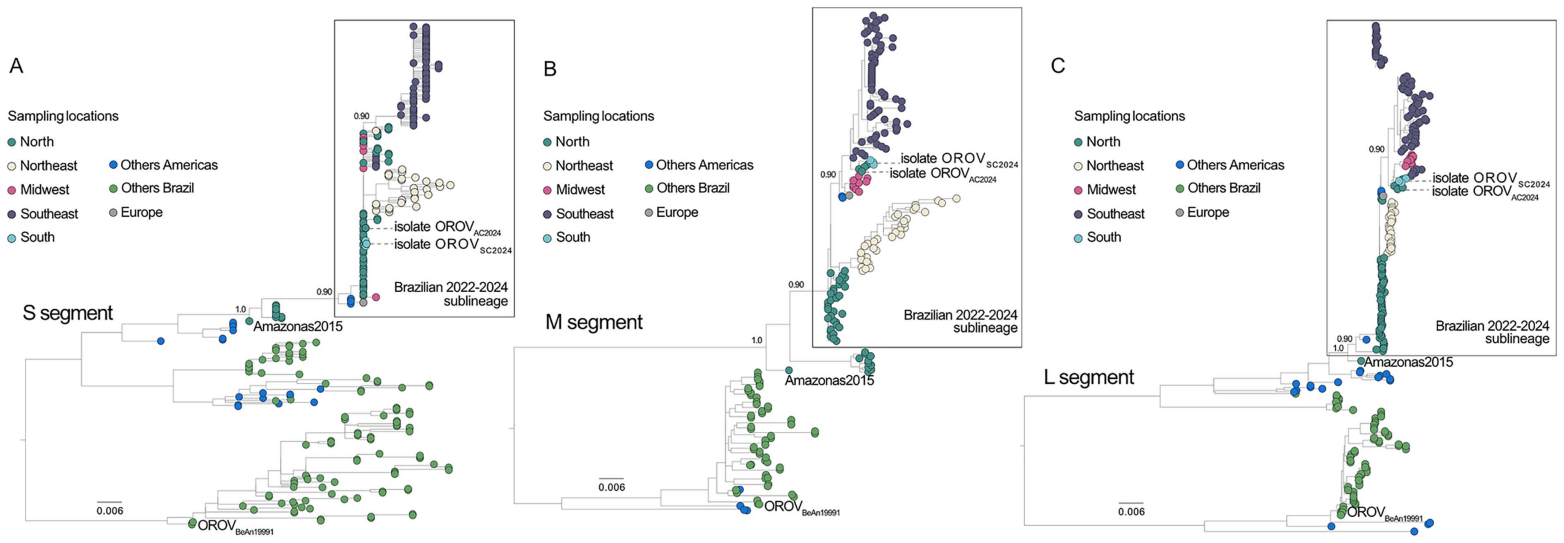
Multi-segment phylogenetic analysis of the Oropouche virus (OROV) isolates. Maximum-likelihood phylogeny of the OROV S **(A)**, M **(B)**, and L **(C)** segments, including representative genomes from Brazil, other regions of the Americas, and Europe. Sequences are colored according to sampling location, and genomes associated with fatal human cases are indicated by outlined symbols. The two reassortant isolates analyzed in this study—OROV_AC2024_ and OROV_SC2024_—are highlighted. The prototype strain OROV_BeAn19991_ is included in all three trees to provide an evolutionary anchor. The 2024 isolates cluster within a well-supported reassortant lineage circulating in Brazil between 2022 and 2024, which is phylogenetically rooted in a 2015 sequence from Tefé, Amazonas. This lineage includes genomes previously associated with severe and fatal clinical outcomes. Node support values are shown for key nodes, and the scale bar indicates nucleotide substitutions per site.

Phylogenetic analysis of the OROV S, M, and L segments was performed using independent datasets to contextualize the two reassortant isolates obtained in 2024 from Acre (OROV_AC2024_, GenBank accession PQ247716, PQ168300, and PQ168431) and Santa Catarina (OROV_SC2024_, PQ247721, PQ168305, and PQ168436) within the broader genetic diversity of OROV circulating in Brazil and the Americas (Figure 2). All datasets included representative genomes from different Brazilian regions, sequences from other American countries, European isolates, genomes previously associated with severe and fatal human cases, and the reference strain BeAn19991 (GenBank accessions KP052850, KP052851.1, and KP052852), which was included as an evolutionary anchor. Both 2024 isolates clustered within a well-supported reassortant lineage that has been circulating in Brazil in recent years, here referred to as the Brazilian 2022–2024 sublineage (de Melo Iani et al., 2025). This clade includes multiple genomes sampled across distinct Brazilian regions and is phylogenetically rooted in a sequence isolated in Tefé, Amazonas, in 2015, consistent with previous reports (Naveca et al., 2024; de Melo Iani et al., 2025). Within this clade, and consistently across all genome segments, the Acre and Santa Catarina isolates do not form a distinct subcluster nor occupy basal or divergent positions relative to other contemporaneously circulating genomes. Instead, they are interspersed among sequences sampled between 2022 and 2024, indicating that they are representative members of an already established reassortant lineage rather than the result of a recent evolutionary divergence. Overall, phylogenetic analysis does not support a distinct evolutionary trajectory or a unique genomic signature for the experimental isolates across segments. Instead, the observed phenotypic differences are consistent with biological properties shared by an established reassortant lineage that has become epidemiologically dominant in Brazil and includes viruses associated with severe clinical outcomes.

### 3.2 Increased pathogenicity of contemporary reassortant OROV linages in the AG129 mouse model

**Figure 3:**
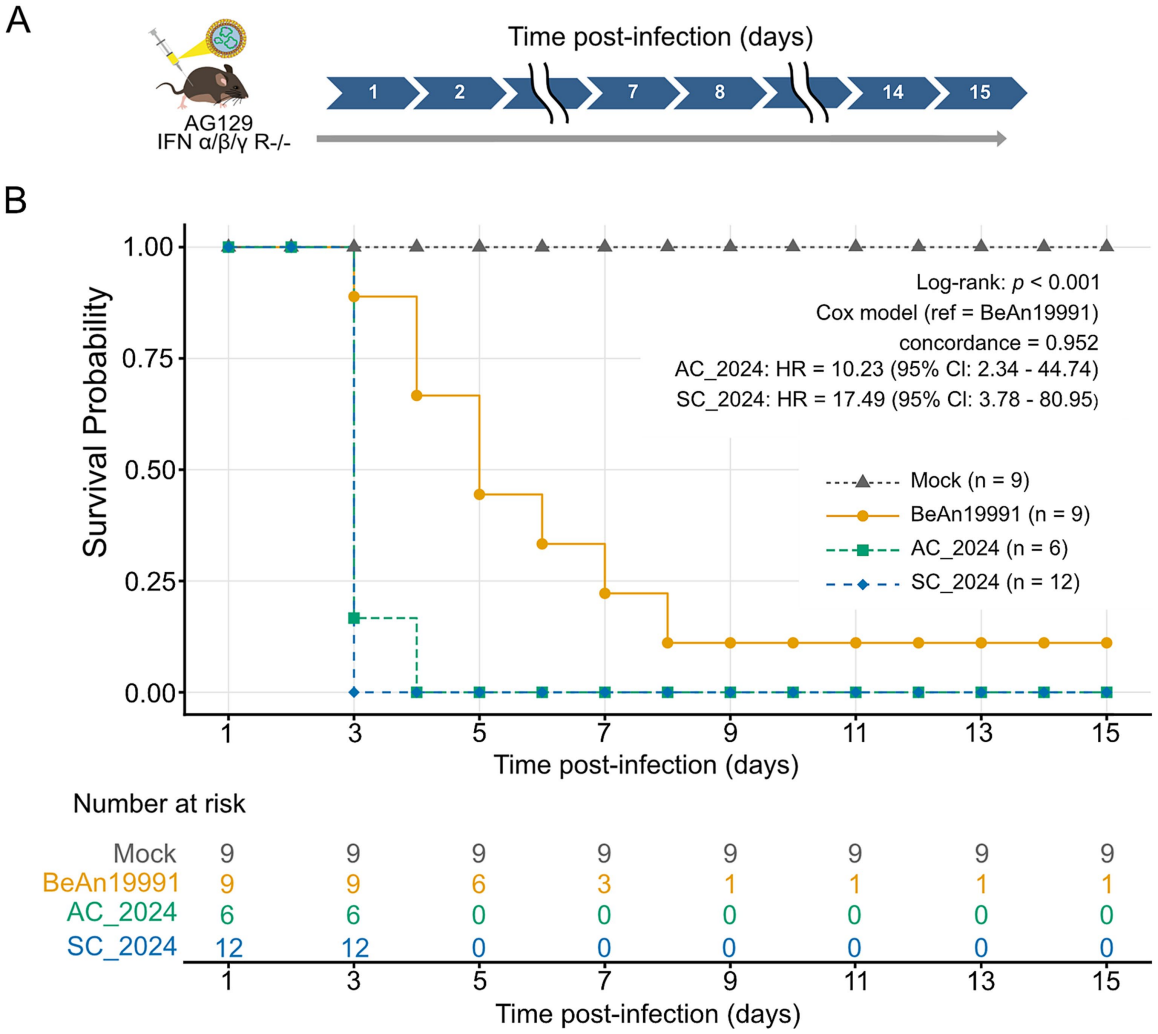
Comparative survival analysis of OROV strains in AG129 mice. **(A)** Schematic overview of the in vivo infection timeline in AG129 mice showing analyzed post-infection time points. **(B)** Survival curve of AG129 mice infected with the ancestral OROV_BeAn19991_ strain or contemporary strains (OROV_AC2024_, OROV_SC2024_) was monitored for 15 days. Survival curves were generated using the Kaplan–Meier method and compared statistically with the Log-Rank test (*p* < 0.0001). The Cox proportional hazards model was used to calculate hazard ratios (HR). The table indicates the number of animals at risk for each time interval.

To determine whether contemporary reassortant OROV lineages exhibit altered virulence *in vivo*, we compared survival outcomes in AG129 mice (IFN- *α*, *β*, *γ* R ^−/−^) following intraperitoneal infection with either the historical prototype strain OROV_BeAn19991_ or the two highly circulating 2024 isolates, OROV_SC2024_ and OROV_AC2024_. Survival was monitored for 15 days post-infection (Figures 3A,B). Mice infected with contemporary isolates experienced rapid and severe disease progression, with complete mortality occurring by 3 days for OROV_SC2024_ and by day 4 for OROV_AC2024_ (Figure 3B). In contrast, infection with the historical OROV_BeAn19991_ strain resulted in a substantially delayed and attenuated disease course, with mortality extending to day 15. All mock-infected controls survived the observation period without clinical signs. Kaplan–Meier analysis confirmed highly significant differences in survival across groups (log-rank test, *p* < 0.001). Cox proportional hazards modelling, using OROV_BeAn19991_ as the reference, revealed dramatically elevated hazard ratios for the contemporary reassortant isolates: OROV_AC2024_: HR = 10.23 (95% CI: 2.34–44.74); OROV_SC2024_: HR = 17.49 (95% CI: 3.78–80.95) (Figure 3B). These results demonstrate that reassortant OROV isolates currently circulating in Brazil are substantially more lethal in the AG129 vertebrate model than the historical prototype strain.

### 3.3 Enhanced viremia in mice infected with contemporary reassortant OROV lineages

**Figure 4:**
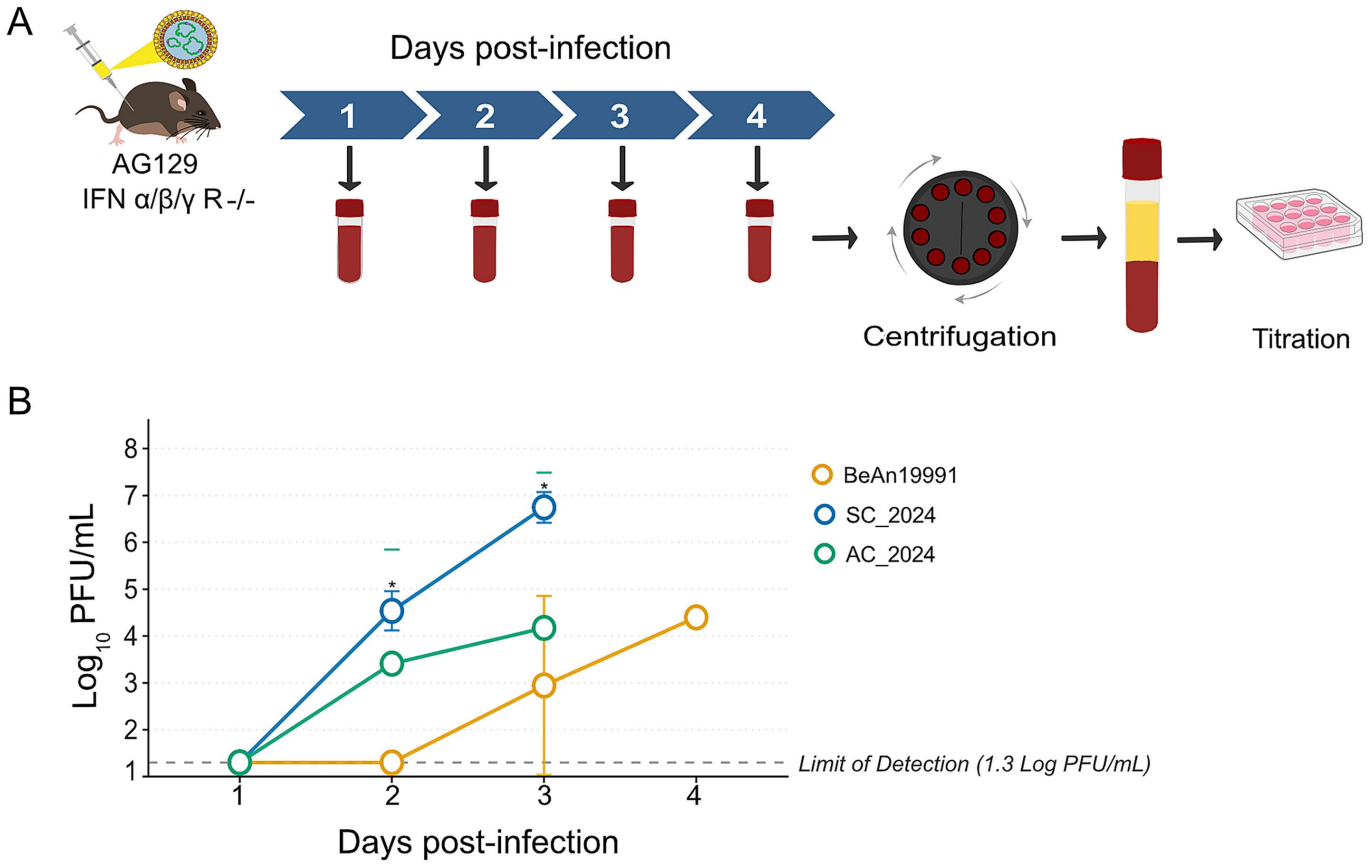
Viral kinetics of OROV in serum of AG129 mice. **(A)** Schematic workflow of sample collection and viral titration in AG129 mice. **(B)** Viral growth curves of OROV_BeAn19991_ prototype strain and contemporary strains (OROV_AC2024_, OROV_SC2024_) in the serum of immunodeficient AG129 mice. Data points represent the mean viral load (PFU/mL) at each day post infection. The horizontal dashed line indicates the limit of detection (LOD) of the plaque assay. Note: No data are shown for Day 4 for the contemporary strains, as all mice in these groups succumbed to infection by this time point. Statistical comparisons between the viral strains were performed using the Generalized Linear Model (GLM) Gamma analysis followed by Turkey’s post-hoc pairwise comparisons. Asterisks indicate statistical significance, where * denotes *p* < 0.05.

To investigate whether the accelerated mortality caused by the contemporary OROV isolates was associated with enhanced systemic viral replication, we quantified circulating viral titers in the serum of AG129 mice over the first four days post-infection (Figures 4A,B). Sampling beyond Day 4 was not possible due to the rapid lethality of OROV_SC2024_ and OROV_AC2024_, which resulted in complete mortality Days 3 and 4 (see Section 3.2). Both contemporary isolates produced substantially higher viremia than the historical OROV_BeAn19991_ prototype (Figure 4B). By Day 2 post-infection, OROV_SC2024_ and OROV_AC2024_ had already reached mean titers of 4.53 and 3.40 log₁₀ PFU/mL, respectively, whereas OROV_BeAn19991_ remained below the limit of detection. Statistical analysis (GLM Gamma with Tukey adjustment) confirmed that OROV_SC2024_ titers were significantly higher than those of OROV_BeAn19991_ at this early time point (*p* = 0.01), while no significant difference was detected between OROV_SC2024_ and OROV_AC2024_ (Supplementary Table 1). Viremia peaked on Day 3, with OROV_SC2024_ reaching 6.74 log₁₀ PFU/mL and OROV_AC2024_ reaching 4.17 log₁₀ PFU/mL. In contrast, OROV_BeAn19991_ titers were substantially lower (2.94 log₁₀ PFU/mL). OROV_SC2024_ remained significantly higher than OROV_BeAn19991_ on Day 3 (*p* = 0.01), whereas OROV_AC2024_ did not differ statistically from either strain (*p* > 0.05). By Day 4, only OROV_BeAn19991_ infected animals remained alive, showing a delayed peak of 4.39 log₁₀ PFU/mL. Taken together, these results demonstrate that contemporary OROV strains establish faster and higher systemic viral replication *in vivo*, with OROV_SC2024_ displaying particularly rapid viremia kinetics. This enhanced systemic burden is consistent with the accelerated disease progression and increased lethality observed in animals infected with contemporary reassortant lineages.

### 3.4 Contemporary reassortant OROV lineages exhibit stronger peripheral tissue tropism and elevated viral loads

**Figure 5:**
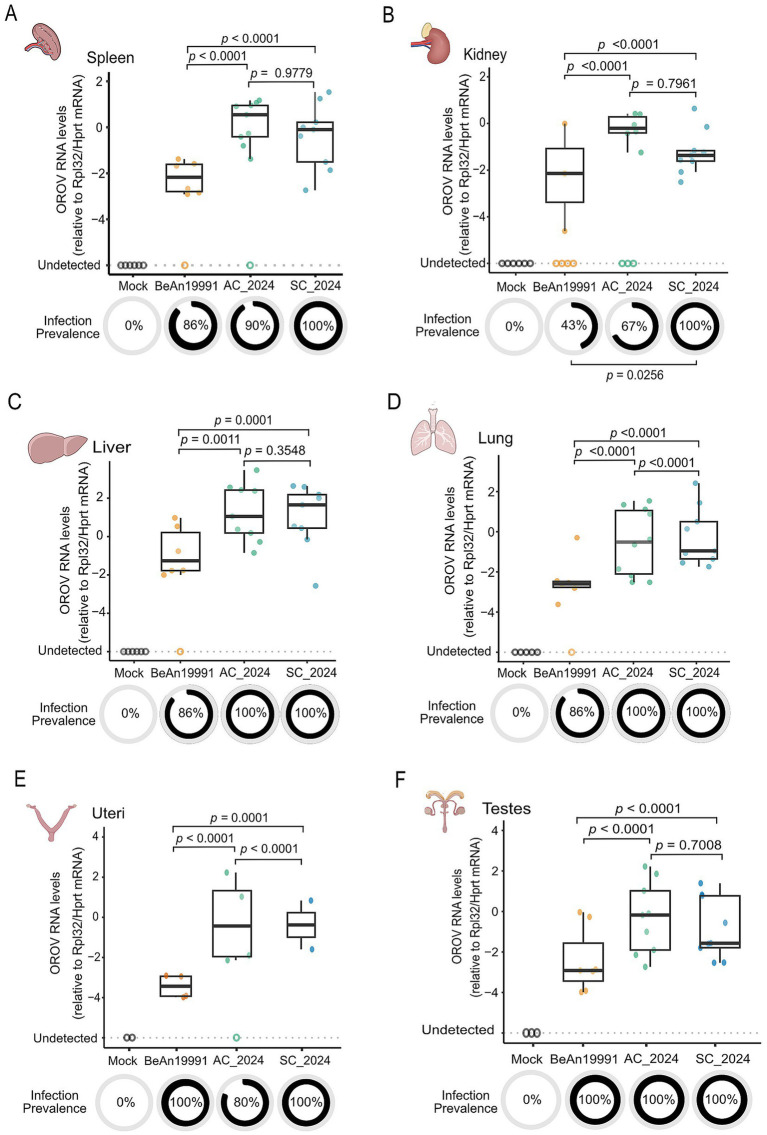
Contemporary OROV strains show enhanced tropism in peripheral tissues. Viral loads of the ancestral (OROV_BeAn19991_) and contemporary (OROV_AC2024_, OROV_SC2024_) strains were quantified by RT-qPCR in various tissues of AG129 mice at 48 h post-infection. Viral RNA levels are expressed as the relative quantity (2^−ΔΔ*Ct*^), normalized to RpL32 and HPRT. Tissues analyzed: **(A)** spleen, **(B)** liver, **(C)** kidney, **(D)** lung, **(E)** uterus, and **(F)** testes. Statistical comparisons between the viral strains were performed using the eneralized inear odel Generalized Linear Model (GLM) Gamma analysis followed by Turkey’s post-hoc pairwise comparisons. Differences were considered significant when *p* < 0.05.

To assess whether the increased viremia observed *in vivo* translated into enhanced systemic dissemination, we quantified viral RNA across major organs at 48 h post-infection—an experimental point selected to ensure inclusion of all strains prior to the rapid mortality of mice infected with contemporary lineages. Tissue viral loads were measured by RT-qPCR (Figure 5). All strains could establish infection in multiple peripheral organs; however, contemporary reassortant isolates (OROV_AC2024_ and OROV_SC2024_) displayed markedly higher viral burdens across nearly all tissues examined. In the spleen and kidney (Figures 5A,B), both contemporary strains reached significantly higher viral loads than the historical OROV_BeAn19991_ prototype (GLM Gamma with Tukey post-hoc; *p* < 0.0001 for all comparisons; Supplementary Table 2), indicating enhanced systemic replication. A similar pattern was observed in the liver (Figure 5C), where OROV_AC2024_ (*p* = 0.0011) and OROV_SC2024_ (*p* = 0.0001) replicated to significantly greater levels than the ancestral strain. Infection prevalence further highlighted these differences: whereas OROV_BeAn19991_ exhibited limited kidney tropism, OROV_SC2024_ infected 100% of animals, representing a significant increase in infection frequency (Fisher’s exact test, *p* = 0.0256), consistent with a broadened or more efficient dissemination capacity. Given clinical signs of respiratory distress and bloody secretion in mice infected with contemporary isolates, we next examined the lung. Both OROV_AC2024_ and OROV_SC2024_ showed significantly elevated pulmonary viral loads relative to OROV_BeAn19991_ (*p* < 0.0001; Figure 5D), supporting a link between increased respiratory tropism and disease severity. Analysis of reproductive tissues revealed a similar trend. In both the uterus and testes (Figures 5E–F), OROV_AC2024_ achieved significantly higher viral loads than OROV_BeAn19991_ (*p* < 0.0001), suggesting a pronounced tropism of this strain for reproductive organs and raising potential implications for sexual or vertical transmission. Together, these results demonstrate that contemporary reassortant OROV lineages display enhanced and more widespread tissue replication compared with the historical prototype, consistent with their increased virulence and accelerated disease progression observed *in vivo*.

### 3.5 Region-specific CNS infection reveals elevated neuroinvasion by contemporary reassortant OROV lineages

**Figure 6:**
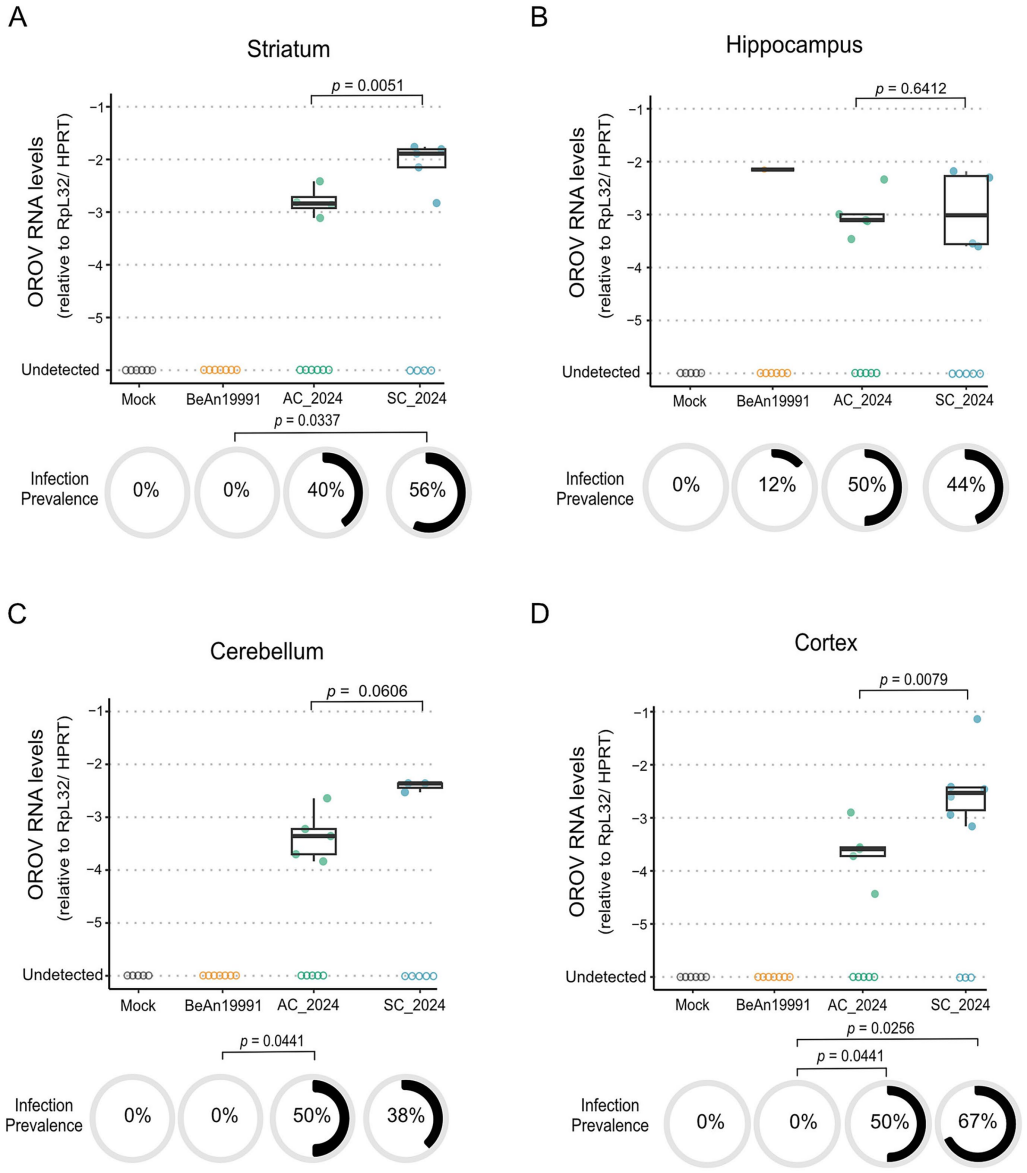
Contemporary OROV strains show enhanced tropism for central nervous system tissues. Viral loads of the ancestral (OROV_BeAn19991_) and contemporary (OROV_AC2024_, OROV_SC2024_) strains were quantified by RT-qPCR in various CNS tissues of AG129 mice at 48 h post-infection. Viral RNA levels are expressed as the relative quantity (2^−ΔΔ*Ct*^), normalized to RpL32 and HPRT. Tissues analyzed: **(A)** striatum, **(B)** hippocampus, **(C)** cerebellum, and **(D)** cortex. Statistical comparisons between the viral strains were performed using the Generalized Linear Model (GLM) Gamma analysis followed by Turkey’s post-hoc pairwise comparisons. Differences were considered significant when *p* < 0.05.

To determine whether the increased virulence of contemporary OROV strains extended to the central nervous system, we quantified viral RNA across four brain regions—striatum, hippocampus, cerebellum, and cortex—48 h post-infection (Figure 6). Contemporary isolates (OROV_AC2024_ and OROV_SC2024_) displayed clear evidence of CNS invasion, whereas neuroinfection by the historical OROV_BeAn19991_ prototype was rare or absent (Figure 6). Neuroinvasion was not uniform across the brain. The striatum and cortex emerged as the most permissive regions, with OROV_SC2024_ exhibiting significantly higher viral RNA levels than OROV_AC2024_ in both tissues (striatum: *p* = 0.0051; cortex: *p* = 0.0079; GLM Gamma with Tukey post-hoc; Supplementary Table 2). In the hippocampus and cerebellum, contemporary isolates were detectable in multiple animals, but viral loads did not differ significantly from the historical strain, reflecting region-specific variability in CNS susceptibility (Figure 6). Strikingly, OROV_BeAn19991_ failed to infect any CNS region except for a single hippocampal sample, highlighting the limited neuroinvasive potential of the historical prototype. In contrast, contemporary isolates showed robust CNS dissemination, with infection prevalence patterns reinforcing their enhanced neurotropism. OROV_SC2024_ infected the striatum (*p* = 0.0337) and cortex (*p* = 0.0256) significantly more frequently than OROV_BeAn19991_, while OROV_AC2024_ exhibited significantly greater infection prevalence in the cerebellum (*p* = 0.0441) and cortex (*p* = 0.0441) (Fisher’s exact test). Together, these findings demonstrate that contemporary reassortant OROV lineages—particularly OROV_SC2024_—possess an increased capacity to cross the blood–brain barrier and establish productive infection in multiple CNS regions. This enhanced neuroinvasive phenotype aligns with the increased lethality and accelerated disease progression observed *in vivo*, suggesting that recent viral evolution has expanded OROV’s CNS tropism and pathogenic potential.

### 3.6 Contemporary reassortant OROV linages selectively induce elevated IL-1β expression in the spleen

**Figure 7:**
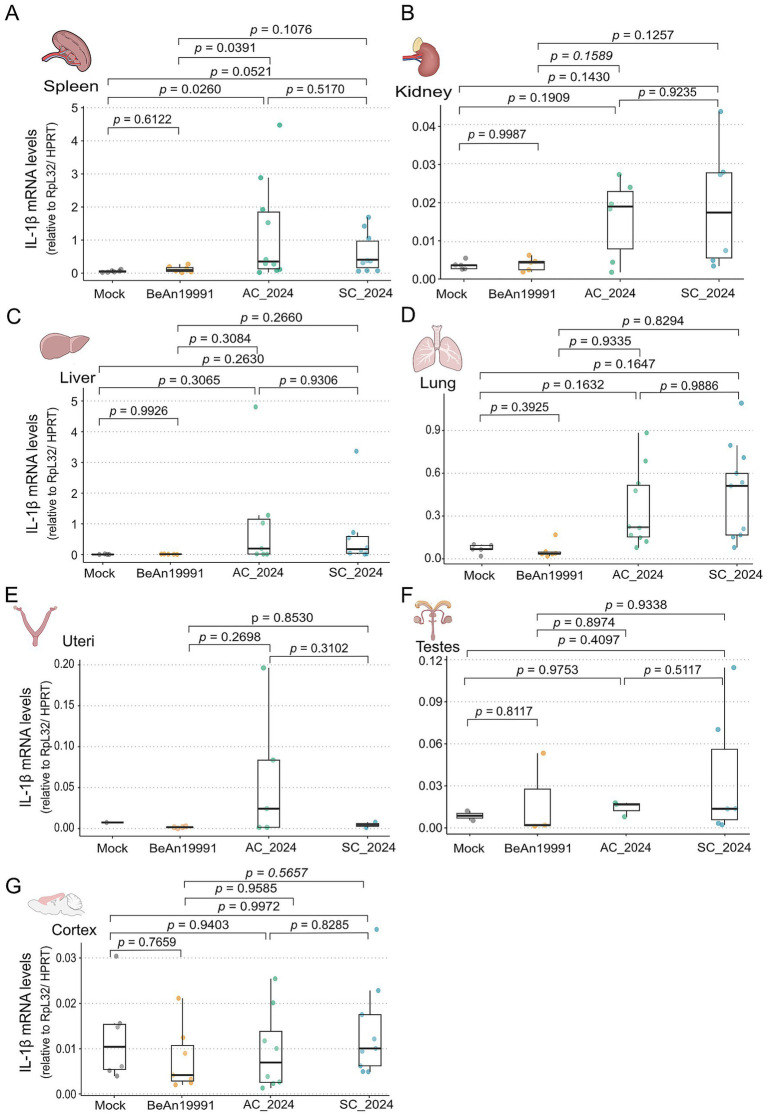
IL-1β expression in infected AG129 tissues. Relative expression of IL-1β quantified by RT-qPCR across various tissues of AG129 mice at 48 h post-infection with OROV strains. The tissues include: **(A)** spleen, **(B)** liver, **(C)** kidney, **(D)** lung, **(E)** uterus, **(F)** testes and **(G)** cortex. Gene RNA levels are expressed as the relative quantity (2^–ΔΔ*Ct*^), normalized to RpL32 and HPRT reference genes. Statistical comparisons between the viral strains were performed using the generalized linear model (GLM) gamma analysis followed by Turkey’s post-hoc pairwise comparisons. Differences were considered significant when *p* < 0.05.

To determine whether the enhanced replication of contemporary OROV lineages was associated with altered inflammatory signalling, we quantified IL-1β, TNF-*α*, and IL-6 mRNA levels across peripheral tissues and the cortex at 48 h post-infection (Figure 7). Cytokine expression was normalized to RpL32/HPRT and analyzed using a GLM Gamma model with Tukey post-hoc comparisons (Supplementary Tables 3). Among all tissues examined, the spleen exhibited the most pronounced cytokine response, with a significant increase in IL-1β expression in mice infected with the OROV_AC2024_ strain relative to Mock controls (*p* = 0.0260) and to the historical OROV_BeAn19991_ prototype (*p* = 0.0391) (Figure 7A). No significant IL-1β upregulation was detected in mice infected with OROV_SC2024_ despite its higher viral burden in this organ. Across the kidney, liver, lung, uteri, and testes, IL-1β levels did not differ significantly among the viral strains or compared with the Mock group (Figures 7B–F), indicating that peripheral cytokine activation was not broadly elevated despite substantial viral replication in these tissues. Consistent with the comparatively modest viral loads detected in the CNS, IL-1β expression in the cortex remained low and statistically indistinguishable across all experimental groups (Figure 7G). Likewise, neither TNF-α or IL-6 expression differed significantly in any tissue evaluated, including the cortex (Supplementary Figures 1, 2; Supplementary Tables 4, 5). Taken together, these findings indicate that contemporary OROV strains do not elicit a generalized inflammatory response but instead induce a selective IL-1β signature in the spleen, particularly following infection with the OROV_AC2024_ strain. This localized cytokine activation may reflect early innate immune sensing at a major site of viral replication rather than widespread systemic inflammation.

## 4 Discussion

Oropouche virus (OROV) is currently undergoing a profound epidemiological transition, reflected by its rapid geographic expansion, escalating case numbers, and an unexpected surge in severe clinical outcomes, including adult fatalities, congenital infections, and neonatal neurological damage (Filho et al., 2024b; Martins-Filho et al., 2024; Schwartz et al., 2024). These shifts in disease burden have coincided with increasingly frequent detections of OROV in non-endemic regions of Brazil and other South American countries, suggesting that the virus is escaping its historical ecological boundaries and establishing new transmission zones across the continent. In parallel, genomic surveillance has revealed a growing diversity of viral reassortants characterized by novel segment combinations and patterns of adaptive mutations (Naveca et al., 2024; de Melo Iani et al., 2025). This expanding genomic heterogeneity raises the possibility that contemporary OROV lineages have acquired enhanced biological traits that influence transmissibility, pathogenicity, and tissue tropism. As recently emphasized in global assessments of arbovirus emergence, predicting epidemic risk requires directly linking viral genetic diversification to measurable phenotypic changes *in vivo* (Ketkar et al., 2019; Hill et al., 2025). The present study addresses this gap by offering the first controlled comparison of ancestral and contemporary human OROV isolates in a vertebrate infection model, while acknowledging that we focused on a limited number of strains and on a highly susceptible mouse background.

Our findings reveal that the contemporary isolates OROV_AC2024_ and OROV_SC2024_ exhibit a dramatic shift toward hyperacute systemic virulence when compared to the historical OROV_BeAn19991_ prototype strain. Both modern strains produced uniformly lethal infections in AG129 mice, with OROV_SC2024_ inducing complete mortality as early as 72 h post-infection. This precipitous disease course correlated with exceptionally high levels of viremia, particularly in OROV_SC2024_, which reached nearly 7 log₁₀ PFU/mL by Day 3—more than double that of the ancestral lineage. Such explosive viral amplification suggests that recent OROV evolution has favored increased replicative fitness *in vivo*, a phenomenon reminiscent of virulence transitions observed in other arboviruses following ecological expansion or reassortment events. For example, single mutations enabling enhanced replication in mosquito vectors or vertebrate hosts have been tied to major epidemic waves of CHIKV, ZIKV, and DENV (Tsetsarkin et al., 2007; Chen et al., 2022; Fan et al., 2024; Ning et al., 2024). At the same time, these comparisons must be interpreted with caution: the OROV_BeAn19991_ strain used here has undergone multiple rounds of passaging since its isolation in 1960, and such *in vitro* passage history may have attenuated its pathogenicity or altered tropism, potentially leading us to underestimate the intrinsic virulence of ancestral OROV relative to contemporary lineages. This is further supported by our multi-segment phylogenetic analysis, which anchors OROV_BeAn19991_ as a genetically distinct ancestral prototype relative to the 2024 reassortant clades.

An additional methodological consideration when comparing historical and contemporary isolates is the possibility that adaptive mutations may arise during *in vitro* amplification, potentially altering viral phenotype. To minimize this risk, the contemporary OROV isolates used in this study were propagated under limited passage conditions, and the viral stocks used for animal inoculation corresponded to low-passage preparations. Furthermore, to directly address the possibility of laboratory-derived variation, we performed high-depth next-generation sequencing on the exact P2 viral stocks used for mouse infection. The consensus sequences obtained were identical to those originally derived from the corresponding patient sera, with no additional fixed nucleotide substitutions detected across the genome. These results indicate that the phenotypic differences observed between the prototype strain and the contemporary isolates are unlikely to reflect mutations acquired during laboratory propagation and instead support the interpretation that they represent intrinsic properties of the currently circulating reassortant lineages.

The evolutionary shift we infer is further reflected in the expanded tissue tropism of the contemporary OROV lineages. Although all isolates disseminated to peripheral organs by 48 h post-infection, modern strains reached substantially higher viral loads in the spleen, liver, lung, kidney, and reproductive tissues compared to OROV_BeAn19991_. The reproductive tropism observed for OROV_AC2024_, with notably elevated viral loads in the testes, is particularly striking. This phenotype aligns closely with recent clinical findings of prolonged OROV RNA detection in semen (Iglói et al., 2025) and documented cases of fetal infection and neonatal brain injury (Martins-Filho et al., 2024). Together, these observations raise the important possibility that contemporary OROV strains possess an enhanced capacity for sexual or vertical transmission—an epidemiological pattern previously noted during the emergence of ZIKV. Future work including additional low-passage archival isolates and a broader panel of contemporary strains will be crucial to determine whether this expanded tissue tropism is a general feature of the 2023–2024 OROV expansion or is restricted to specific lineages such as OROV_AC2024_.

Comparative evidence from previous *in vivo* studies reinforces the conclusion that the historical OROV_BeAn19991_ prototype exhibits an attenuated and delayed pathogenic profile relative to the contemporary lineages characterized here. Earlier work in newborn BALB/c mice showed that OROV_BeAn19991_ causes a slowly progressive lethal infection, with mortality beginning only after Day 5 and some animals surviving until Day 15 (Santos et al., 2012). Similarly, Proenca-Modena et al. (2015) demonstrated that disease severity is strongly dependent on host immunogenetics: while 3-day-old C57BL/6 wild-type pups exhibited 66.6% mortality following OROV_BeAn19991_ infection, immunocompetent mice aged 3–9 weeks displayed complete resistance, underscoring the critical role of intact type I interferon signalling in restricting OROV replication. Studies in interferon-deficient models further highlight this dependency. De Mendonça et al. (2021), using AG129 mice, also observed mortality after OROV_BeAn19991_ infection, but viral replication remained modest, with peak viremia at Day 5 not exceeding 6 log₁₀ PFU/mL. This pattern contrasts sharply with the explosive systemic replication observed for contemporary reassortant strains in our study, which reached similar or higher titers by Day 3 alone. Taken together, these previous investigations consistently portray OROV_BeAn19991_ as a virus with limited intrinsic virulence and delayed replication kinetics, even in highly susceptible hosts. Our findings therefore demonstrate that the phenotypic behavior of modern reassortant isolates cannot be explained by host susceptibility alone; instead, they point to genuine evolutionary changes that have increased replicative fitness, accelerated disease progression, and expanded tissue tropism relative to the ancestral lineage. Nonetheless, because AG129 mice lack both type I and type II interferon signalling, the absolute magnitude of these differences may not fully reflect what occurs in immunocompetent hosts, reinforcing the need for complementary studies in partially interferon-competent models, non-human primates, or human organoid systems.

The most compelling evidence of altered viral biology, however, lies in the pronounced neuroinvasive capability of the contemporary strains. Whereas the OROV_BeAn19991_ prototype showed minimal CNS infection in AG129 mice, both modern isolates—particularly OROV_SC2024_—disseminated efficiently to the cortex, hippocampus, striatum, and cerebellum, achieving viral loads comparable to those seen in peripheral organs. This robust neurotropism provides an experimental correlate for the increasing reports of neurological disease in human OROV infections, including meningoencephalitis and fatal congenital brain injury, where OROV RNA has been detected in cerebrospinal fluid and multiple brain regions (Filho et al., 2024a; Martins et al., 2024). These findings strongly suggest that reassortment-driven genomic changes have expanded the anatomical niche of OROV within the vertebrate host, enhancing its ability to breach the blood–brain barrier and replicate within the CNS. Given that our conclusions are based on two contemporary reassortant strains, a broader sampling of the current genomic diversity will be important to determine whether enhanced neuroinvasion is widespread among circulating lineages or concentrated in a subset of emergent genotypes such as OROV_SC2024_.

Interestingly, despite the rapid systemic infection and overwhelming viral loads, cytokine profiling revealed a surprisingly localized inflammatory response. IL-1β expression was significantly upregulated only in the spleen—predominantly following OROV_AC2024_ infection—whereas IL-6 and TNF-*α* levels remained unchanged across tissues. This restricted cytokine signature contrasts with clinical reports of severe OROV disease, where elevated IL-6 levels and multisystem organ failure are frequently observed (Bandeira et al., 2024; Có et al., 2025). The divergence likely reflects the early time point examined in our study: by necessity, cytokine measurements were performed at 48 h post-infection to precede the hyperacute mortality of contemporary strains, potentially missing later inflammatory cascades that contribute to severe disease in humans. Thus, our data suggest that in AG129 mice early mortality is driven more by rapid viral replication and organ-specific tropism than by an overt cytokine storm, but do not exclude the possibility that dysregulated inflammation emerges at later stages.

Integrating these findings reveals a coherent model that links recent OROV evolution to its changing epidemiological profile. Contemporary lineages have acquired genomic alterations—including reassortments—associated with enhanced replication and systemic dissemination. These phenotypes translate into increased neurovirulence and reproductive tissue involvement, aligning with the rise in severe neurological and congenital disease. Furthermore, the rapid expansion of OROV into non-endemic regions of Brazil and neighbouring countries may be facilitated by these evolved traits, combined with ecological changes affecting vector distribution. This genotype–phenotype–epidemiology linkage mirrors patterns observed in other rapidly emerging arboviruses and underscores the adaptive potential of segmented RNA viruses like OROV. At the same time, the restricted number of contemporary isolates analysed, and the use of a single, highly susceptible host genotype mean that our model should be viewed as a working framework to be tested and refined, rather than as a definitive description of all circulating OROV lineages.

These insights carry several important implications for public health and future research. First, high-resolution genomic surveillance is urgently needed to identify reassortant strains and specific mutations associated with increased virulence or neurotropism. Second, given the strong reproductive tropism observed in our model and in clinical cases, systematic evaluation of sexual and vertical transmission potential is warranted. Third, the development of antivirals and vaccines against OROV must incorporate contemporary strains whose biological behavior differs fundamentally from historical prototypes commonly used in laboratory settings—particularly those that may have been attenuated by extensive *in vitro* passaging. Finally, more refined animal models—especially those with partial interferon competence—and human-relevant systems such as organoids will be essential to dissect the host pathways that govern susceptibility, CNS invasion, and severe disease and to validate the generality of the phenotypes reported here.

In summary, our experimental data provide compelling evidence that OROV is undergoing functional evolutionary changes that may contribute to increased pathogenicity, broader tissue tropism, and enhanced neuroinvasion. These phenotypic patterns provide a potential mechanistic context for the rising clinical severity and expanding geographic distribution documented during the 2023–2024 outbreaks. As emphasized in prior analyses of emerging arboviruses, anticipating epidemic trajectories requires recognizing when viral evolution can influence disease biology (Weaver and Barrett, 2004; Holmes et al., 2016; Weaver et al., 2018). The evidence presented here suggests that Oropouche virus should be considered a pathogen of growing public health relevance in the Americas and underscores the need for strengthened surveillance, continued mechanistic investigation, and the development of effective countermeasures—while acknowledging that important uncertainties remain regarding viral diversity, host responses, and long-term transmission dynamics.

## Data Availability

The datasets presented in this study can be found in online repositories. The names of the repository/repositories and accession number(s) can be found in the article/Supplementary material.
