# Supplementary figures and images for: Enhanced virulence and neuroinvasion of contemporary Oropouche virus strains in the AG129 mouse model
Source: Front Microbiol. 2026 May 19;17:1771021. doi: 10.3389/fmicb.2026.1771021 (PMC13226609; doi:10.3389/fmicb.2026.1771021)

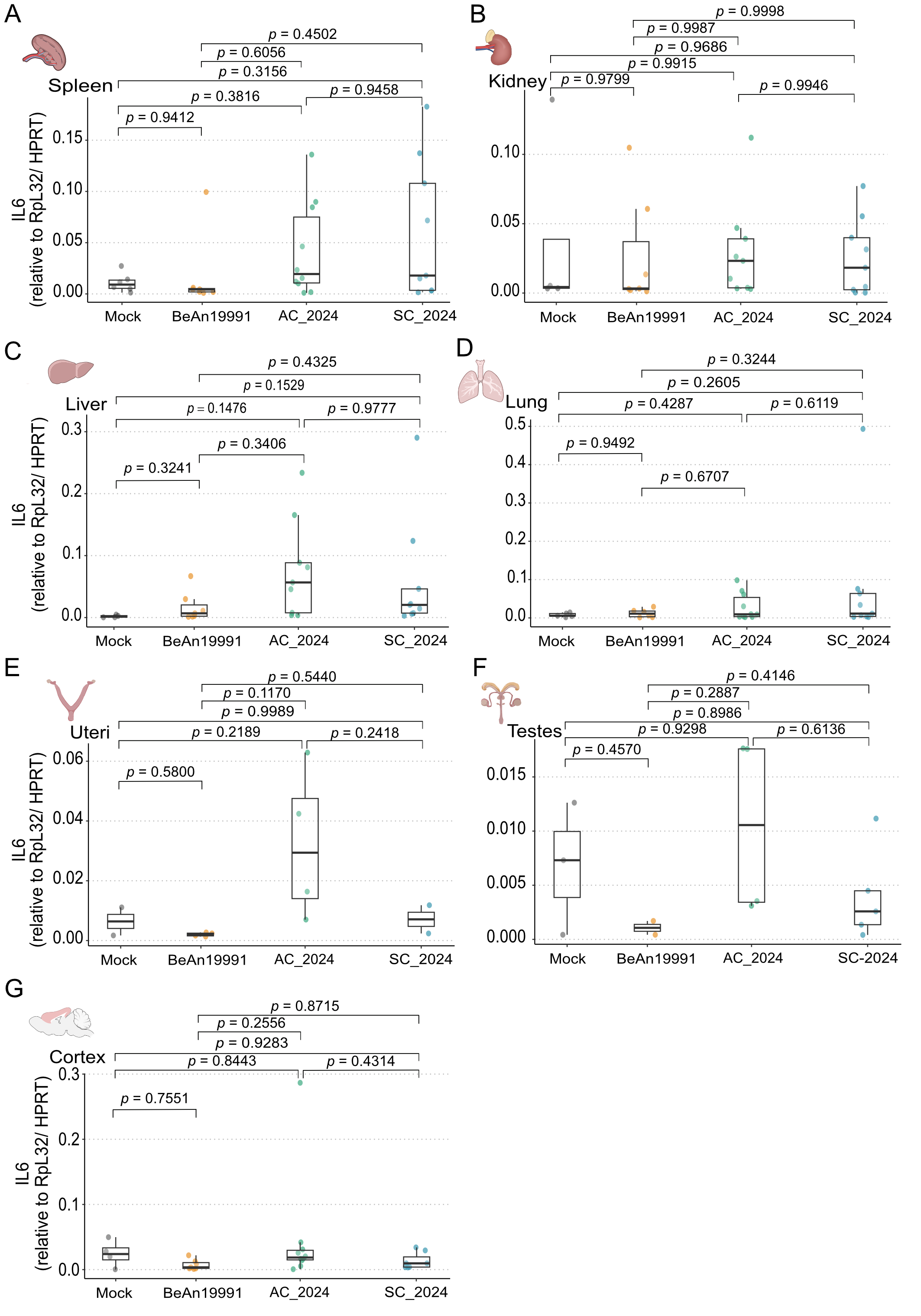

Supplement: Supplementary file 1 [file Image_1.tiff]

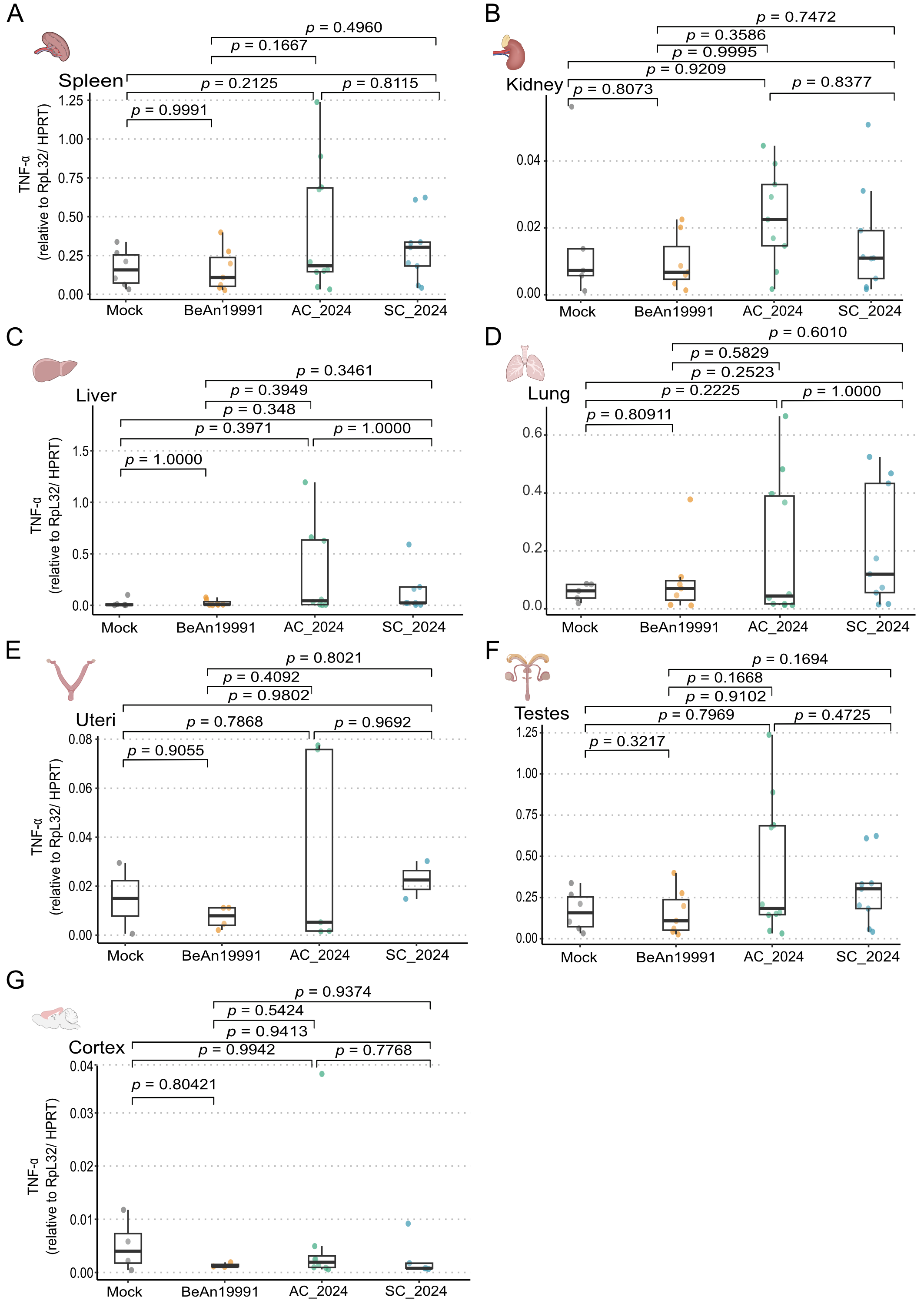

Supplement: Supplementary file 2 [file Image_2.tiff]
