# Supplementary material for: Enhanced virulence and neuroinvasion of contemporary Oropouche virus strains in the AG129 mouse model
Source: Front Microbiol. 2026 May 19;17:1771021. doi: 10.3389/fmicb.2026.1771021 (PMC13226609; doi:10.3389/fmicb.2026.1771021)
Supplement: Supplementary file 3 [file Supplementary_file_1.docx]

Supplementary Material

**Supplementary Figure 1.** **IL-6 Expression in Infected AG129 Tissues.** Relative expression of IL-6 quantified by RT-qPCR across various tissues of AG129 mice at 48 hours post-infection with OROV strains. The tissues include: (A) spleen, (B) kidney, (C) liver, (D) lung, (E) uteri (F) testes e (G) cortex. Gene RNA levels are expressed as the relative quantity (2^–ΔΔ^*^Ct^*), normalized to RPL32 and HPRT reference genes. Data were analyzed by the Kruskal-Wallis test with Dunn's post-hoc test for pairwise comparisons. Differences were considered significant when *p* < 0.05. The exact *p*-values for all pairwise comparisons are indicated above the respective bars.

**Supplementary Figure 2.** **TNF-α Expression in Infected AG129 Tissues.** Relative expression of TNF- α quantified by RT-qPCR across various tissues of AG129 mice at 48 hours post-infection with OROV strains. The tissues include: (A) spleen, (B) kidney, (C) liver, (D) lung, (E) uteri (F) testes e (G) cortex. Gene RNA levels are expressed as the relative quantity (2^–ΔΔ^*^Ct^*), normalized to RPL32 and HPRT reference genes. Data were analyzed by the Kruskal-Wallis test with Dunn's post-hoc test for pairwise comparisons. Differences were considered significant when *p* < 0.05. The exact *p*-values for all pairwise comparisons are indicated above the respective bars.

**Supplementary Table 1. Statistical Analysis of OROV Viral Load in Mouse Serum.** Statistical analysis of OROV viral load in the serum of AG129 mice. The analysis was performed using the Generalized Linear Model (GLM) with Gamma distribution followed by Tukey’s post-hoc pairwise comparisons to determine significant differences between viral strains at each day post-infection. Note: Statistical comparisons were restricted to Day 2 and Day 3 post-infection, as viral load was not detectable in any experimental group on Day 1. The full analysis details the significant differences between viral strains at these specific time points.

**Supplementary Table 2. Statistical Analysis of OROV Viral Load in Tissue.** Statistical analysis of OROV viral load in various tissues of AG129 mice. The viral load quantification was performed at a single time point: 48 hours post-infection. The analysis was conducted using the Generalized Linear Model (GLM) with Gamma distribution followed by Tukey’s post-hoc pairwise comparisons to determine significant differences between viral strains across each specific tissue analyzed (spleen, liver, kidney, lung, uterus, testes, cortex, striatum, hippocampus, and cerebellum). The full analysis details the significant differences between viral strains within each specific tissue.

**Supplementary Table 3. Statistical Analysis of IL-1β** **Expression in Infected AG129 Tissues.** Statistical analysis of IL-1β expressions in various tissues of AG129 mice. The analysis was performed on RT-qPCR data collected at a single point: 48 hours post-infection. The analysis was conducted using the Generalized Linear Model (GLM) with Gamma distribution followed by Tukey’s post-hoc pairwise comparisons to determine significant differences between viral strains and control groups across each specific tissue analyzed (spleen, liver, kidney, lung, uterus, testes, cortex, striatum, hippocampus, and cerebellum). The full analysis details the significant differences in IL-1β expression between viral strains within each specific tissue.

**Supplementary Table 4. Statistical Analysis of IL-6 Expression in Infected AG129 Tissues.** Statistical analysis of IL-6 expressions in various tissues of AG129 mice. The analysis was performed on RT-qPCR data collected at a single point: 48 hours post-infection. The analysis was conducted using the Generalized Linear Model (GLM) with Gamma distribution followed by Tukey’s post-hoc pairwise comparisons to determine significant differences between viral strains and control groups across each specific tissue analyzed (spleen, liver, kidney, lung, uterus, testes, cortex, striatum, hippocampus, and cerebellum). The full analysis details the significant differences in IL-6 expression between viral strains within each specific tissue.

**Supplementary Table 5. Statistical Analysis of TNF-α Expression in Infected AG129 Tissues.** Statistical analysis of TNF-α expressions in various tissues of AG129 mice. The analysis was performed on RT-qPCR data collected at a single point: 48 hours post-infection. The analysis was conducted using the Generalized Linear Model (GLM) with Gamma distribution followed by Tukey’s post-hoc pairwise comparisons to determine significant differences between viral strains and control groups across each specific tissue analyzed (spleen, liver, kidney, lung, uterus, testes, cortex, striatum, hippocampus, and cerebellum). The full analysis details the significant differences in TNF-α expression between viral strains within each specific tissue.

.
